# Prevalence and associated factors of sarcopenia in community-dwelling older adults at risk of malnutrition

**DOI:** 10.1186/s12877-022-03704-1

**Published:** 2022-12-24

**Authors:** Samuel Teong Huang Chew, Siew Ling Tey, Menaka Yalawar, Zhongyuan Liu, Geraldine Baggs, Choon How How, Magdalin Cheong, Wai Leng Chow, Yen Ling Low, Dieu Thi Thu Huynh, Ngiap Chuan Tan

**Affiliations:** 1 Department of Geriatric Medicine, Changi General Hospital, Singapore, Singapore; 2 Abbott Nutrition Research and Development, Asia-Pacific Center, Singapore, Singapore; 3 Statistical Services, Cognizant Technologies Solution Pvt. Ltd., Bangalore, India; 4 Abbott Nutrition Research and Development, Columbus, OH USA; 5 Care and Health Integration, Changi General Hospital, Singapore, Singapore; 6 SingHealth-Duke NUS Family Medicine Academic Clinical Program, Singapore, Singapore; 7 Department of Dietetic & Food Services, Changi General Hospital, Singapore, Singapore; 8 Health Services Research, Changi General Hospital, Singapore, Singapore; 9 SingHealth Polyclinics, Singapore, Singapore

**Keywords:** Sarcopenia, Malnutrition, Older adults, Community-dwelling, Prevalence

## Abstract

**Background:**

There is an increasingly strong association between sarcopenia and malnutrition in research findings. We aimed to determine the prevalence and factors associated with sarcopenia in community-dwelling older adults (≥ 65 years) at risk of malnutrition based on Malnutrition Universal Screening Tool (MUST).

**Methods:**

This was a cross-sectional study of 811 participants. Participants were recruited from the general population, community centers, senior activity centers, polyclinics, and hospital. Community-dwelling older adults at risk of malnutrition participated in the study. Participants’ data and measurements were collected at the baseline visit. Data included socio-demographic information, anthropometric measurements, body composition, dietary intakes, and functional assessments. Sarcopenia was defined using the Asian Working Group for Sarcopenia 2019 consensus.

**Results:**

Of the 694 participants with complete datasets, overall prevalence of sarcopenia was 76% (*n* = 530); 57% (*n* = 393) had severe sarcopenia. In the overall cohort, compared to participants without sarcopenia, those with sarcopenia were older, had lower physical activity scale for the elderly score, leg strength, handgrip endurance, mid-upper arm circumference, calf circumference, and bone mass, and had lower dietary protein intake and poorer nutritional status (all *p* ≤ 0.015). After adjusting for confounders, sarcopenia was significantly associated with older age, male gender, higher risk of malnutrition, lower calf circumference, and lower bone mass (all *p* ≤ 0.044).

**Conclusions:**

In community-dwelling older adults at risk of malnutrition, there is a high prevalence of sarcopenia and severe sarcopenia. As such, screening positive for either malnutrition risk or sarcopenia in older adults should prompt screening for the other risk factor, to allow early institution of disease modifying interventions to forestall adverse outcomes for both malnutrition and sarcopenia.

**Trial registration:**

The study was registered at clinicaltrials.gov as NCT03245047.

## Background

Globally, the proportion of older adults (≥ 65 years) increased from 6% in 1990 to 9% in 2019 [1]. This trend is led by Eastern and Southeastern Asia where the proportion of older adults has nearly doubled from 1990 to 2019 (6 to 11%) and is projected to double again by 2050 [1]. With aging comes increased risk for sarcopenia, a condition defined *as age-related loss of muscle mass plus low muscle strength, and/or reduced physical performance* [2].

Sarcopenia is common among older adults worldwide, although its prevalence varies widely by population, by care setting, and by diagnostic criteria used [3–7]. Asian studies report sarcopenia prevalence ranging from 18 to 41% in community-living older adults [3, 8, 9]. In Singapore, the prevalence of sarcopenia in community-dwelling older adults ranges from 23 to 46% [10–12].

Many factors have been reported to be associated with sarcopenia in community-dwelling older adults. Older age [8, 13] and low physical activity level [13] have been associated with sarcopenia. Anthropometric and physical measurements including low body mass index (BMI) [9, 14], small calf circumference [15], and low bone mass [16, 17] have been linked to sarcopenia. Disease conditions such as diabetes [18, 19], chronic obstructive pulmonary disease (COPD) [20], and cognitive impairment [21] have also been correlated with sarcopenia.

Among aging adults, malnutrition is likewise common and leads to adverse functional and clinical outcomes. A recent study conducted among community-dwelling older adults in Singapore found that 31.4% of participants were at risk of malnutrition and 3.9% were malnourished [22]. Older adults who were malnourished or at risk of malnutrition have higher risk of falls compared to those who were well-nourished [23]. Poor nutritional status also increases risk for disability and poorer quality of life in this cohort [24]. Malnutrition is also associated with increased risks of infection, frailty, and mortality [25–27].

Malnutrition and sarcopenia commonly overlap in older people [11, 27–29]. Studies have found that older adults with malnutrition or risk of malnutrition were at higher risk of sarcopenia compared with those of normal nutritional status. The odds ratio for sarcopenia ranges from 9.9 to 13.6 in the malnourished or at-risk groups [11, 29]. In addition, a cohort study over 4 years revealed a four-fold higher risk of developing sarcopenia in malnourished older adults compared to well-nourished older adults [28]. Notably, a team of multidisciplinary experts in Singapore made a consensus recommendation that all older adults should be screened early for muscle impairment and further treated, when needed, before sarcopenia becomes established and lead to adverse outcomes [30].

Therefore, the objectives of the present study were: (i) to determine the prevalence of sarcopenia and its components in community-dwelling older adults (≥ 65 years) who were at risk of malnutrition, and (ii) to identify factors that are associated with sarcopenia.

## Methods

### Study design and participants

Data for this analysis were collected as part of the Strengthening Health In ELDerly through nutrition (SHIELD) study, which involved community-dwelling older people at risk of malnutrition in Singapore. Data from the baseline visit were used in this cross-sectional study. Participants were recruited sequentially between August 2017 and March 2019. Full details of the SHIELD study design have been previously reported [31]. Briefly, participants were recruited based on the following inclusion criteria: males or females aged ≥ 65 years, community-ambulant with or without aid, and at medium- or high-risk of malnutrition using Malnutrition Universal Screening Tool (MUST) [32]. Study participants were community-dwelling (not residing in an intermediate or long-term care services institution) or were discharged directly to home after hospitalization. Individuals with stable chronic disease(s) were eligible. Stable, chronic disease was defined as a long-term condition treatable by regular medication such that symptoms could be controlled to those experienced by the participant when he or she was well.

Eligible participants could consume food and beverages orally, communicate, and follow instructions. Individuals were excluded if they had any of the following conditions: allergy or intolerance to milk products, dementia, type 1 or type 2 diabetes, an active infectious disease (e.g., tuberculosis, Hepatitis B or C, HIV infection), a severe gastrointestinal disorder (e.g., celiac disease, short bowel syndrome, pancreatic insufficiency), cystic fibrosis, end-stage organ failure, pre-terminal disease, acute myocardial infarction within the last 30 days, or active malignancy in the last 5 years.

A total of 811 eligible participants took part in this study. The study was approved by the SingHealth Centralized Institutional Review Board in Singapore, reference number 2017/2273. All participants gave written informed consent. The study was registered at clinicaltrials.gov as NCT03245047.

### Study measurements

At the baseline study visit, a medical history was taken, and sarcopenia-related measurements were done. In addition, socio-demographic information, co-morbidities, dietary intakes, anthropometric measurements, body composition, and functional assessments were collected.

Socio-demographic data including age, gender, ethnicity, marital status, education, number of prescribed drugs, housing status, smoking status, and alcohol consumption were collected during the visit. The Charlson Comorbidity Index (CCI) was used to determine the comorbidity level based on the number and severity of comorbidities [33]. Energy and macronutrient intakes were estimated using 24-hour dietary recall.

Anthropometric measurements included height, mid-upper arm circumference (MUAC), and calf circumference. Standing height was measured using a stadiometer (Avamech B1000). Mid-upper arm circumference was measured at mid-point of the acromion and olecranon. Calf circumference was measured at the largest part of the calf.

Body weight and composition were determined using a bioelectrical impedance analysis (BIA) machine (Tanita MC-780). Body composition measurements included appendicular skeletal muscle mass (ASMM), fat mass, and bone mass. Appendicular skeletal muscle mass index (ASMI) was calculated by dividing appendicular skeletal muscle mass (ASMM) in kilograms (kg) by height in meters (m) squared (kg/m^2^).

Functional assessments were also measured during the study visit. Each participant’s physical activity level was determined using the Physical Activity Scale for the Elderly (PASE) [34]. The Modified Barthel Index (MBI) was used to measure functional independence for 10 activities of daily living [35]. Handgrip strength was measured using a hand-held dynamometer (DynX). Handgrip endurance was measured by asking participants to hold the hand-held dynamometer as long as possible at half-maximal voluntary contraction. Leg strength was determined by measuring isometric knee extension (Lafayette 01165). Physical performance was measured by the Short Physical Performance Battery (SPPB) consisting of three components, which includes the balance test, 4-m gait speed, and 5-time chair stand test [36].

### Sarcopenia diagnosis

Diagnosis of sarcopenia was based on criteria from the updated consensus by the Asian Working Group for Sarcopenia 2019 [2]. *Possible sarcopenia* was defined by low muscle strength or low physical performance. *Sarcopenia* was defined by low ASMI plus low muscle strength or by low ASMI plus low physical performance. *Severe sarcopenia* was defined by low ASMI plus low muscle strength plus low physical performance.

Low ASMI was based on BIA gender-specific cut-off values (male < 7.0 kg/m^2^, female < 5.7 kg/m^2^). Handgrip strength was used to determine muscle strength; low muscle strength was identified by gender-specific handgrip strength cut-off values: male < 28 kg, female < 18 kg. The 5-time chair stand test was used to determine physical performance as a surrogate marker for gait speed [37]; low physical performance was determined by 5-time chair stand test with a cut-off value ≥ 12 seconds.

### Statistical analysis

Data were presented as mean and standard error for continuous variables and as numbers and percentages for categorical variables. Continuous variables were compared between the groups (gender, age, and sarcopenia status) using analysis of variance and categorical variables were compared using Chi-square test or Fisher’s exact test.

Univariate logistic regression was used to examine the associations between sarcopenia and each potential variable. Multiple logistic regression was used to examine the associations between sarcopenia; potential variables were identified based on the literature in this area and on clinical relevance [2, 12, 20, 21, 38–40]. These variables include factors such as age, gender, ethnicity, education level, smoking and drinking status, malnutrition risk, calf circumference, bone mass, and PASE score. Multiple logistic regression analysis was applied when there was a single dichotomous outcome (sarcopenia versus no sarcopenia) and more than one independent variable. Results of both univariate and multiple logistic regression were reported as odds ratios with 95% confidence interval (CI). SAS version 9.4 (SAS Institute, Cary, North Carolina, USA) was used for all statistical analyses. *P* < 0.05 was considered statistically significant.

## Results

**Fig. 1:**
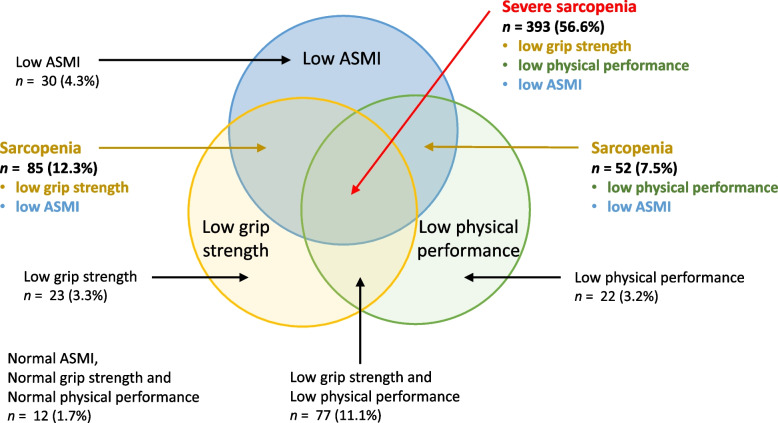
Prevalence of sarcopenia and its components

Of the original 811 community-dwelling older adults (≥ 65 years) who were identified to be at risk of malnutrition and met criteria for inclusion in the analysis, 117 were excluded due to missing data, i.e., 105 without one sarcopenia measurement, 11 without two sarcopenia measurements, and one participant without three sarcopenia measurements. Thus, 694 participants were included in the analysis. The overall prevalence of possible sarcopenia was 83% (*n* = 578), sarcopenia was 76% (*n* = 530), 57% (*n* = 393) of the cohort had severe sarcopenia, and only 1.7% (*n* = 12) had normal ASMI, normal grip strength, and normal physical performance (Table 1 and Fig. 1). By individual sarcopenia criterion, 81% of the study participants had low ASMI, 83% had low handgrip strength, and 78% had low physical performance (Table 1).

**Table 1:** Muscle mass, muscle strength, and physical performance of participants

Variable, ***unit***	All participants (***n*** = 694)	Gender			Age category		
		Female (***n*** = 429)	Male (***n*** = 265)	***p*** value between genders	65 to < 75 years old (***n*** = 425)	≥ 75 years old (***n*** = 269)	***p*** value between age categories
Low ASMI, *n* (%)							
Yes	560 (81)	337 (79)	223 (84)	0.070	333 (78)	227 (84)	0.050
No	134 (19)	92 (21)	42 (16)		92 (22)	42 (16)	
Low handgrip strength, *n* (%)							
Yes	578 (83)	363 (85)	215 (81)	0.232	327 (77)	251 (93)	< 0.001
No	116 (17)	66 (15)	50 (19)		98 (23)	18 (7)	
5-time chair stand test ≥ 12 s, *n* (%)							
Yes	544 (78)	332 (77)	212 (80)	0.417	311 (73)	233 (87)	< 0.001
No	150 (22)	97 (23)	53 (20)		114 (27)	36 (14)	
Sarcopenia, *n* (%)							
Yes	530 (76)	321 (75)	209 (79)	0.223	308 (73)	222 (83)	0.002
No	164 (24)	108 (25)	56 (21)		117 (27)	47 (17)	
Severe sarcopenia, *n* (%)							
Yes	393 (57)	237 (55)	156(60)	0.349	203 (48)	190 (71)	< 0.001
No	301 (43)	192 (45)	109 (41)		222 (52)	79 (29)	
ASMI (kg/m^2^)	5.73 ± 0.03	5.32 ± 0.02	6.39 ± 0.04	< 0.001	5.76 ± 0.04	5.68 ± 0.05	0.196
Handgrip strength (kg)	17.4 ± 0.2	14.3 ± 0.2	22.3 ± 0.4	< 0.001	18.3 ± 0.3	16.0 ± 0.4	< 0.001
Handgrip endurance (s)	71.5 ± 2.0	70.7 ± 2.6	72.8 ± 3.0	0.616	76.6 ± 2.6	63.5 ± 2.9	0.001
Leg strength (kg)	12.0 ± 0.2	11.2 ± 0.2	13.4 ± 0.3	< 0.001	12.5 ± 0.2	11.4 ± 0.3	0.002
SPPB score	9.2 ± 0.1	9.2 ± 0.1	9.3 ± 0.1	0.707	9.8 ± 0.1	8.3 ± 0.1	< 0.001
4-m usual gait speed (m/s)	0.87 ± 0.01	0.87 ± 0.01	0.88 ± 0.01	0.458	0.94 ± 0.01	0.76 ± 0.02	< 0.001
5-time chair stand test duration (s)	15.7 ± 0.2	15.7 ± 0.2	15.8 ± 0.3	0.706	14.7 ± 0.2	17.3 ± 0.4	< 0.001

Note: For continuous variables, mean ± standard error is presented; for categorical variables, n (%) is presented. The sample sizes for some variables are less than the overall stated sample sizes

There were significant differences between the sarcopenia group and the non-sarcopenic group in the overall cohort, in females, and in males (Table 2). Participants with sarcopenia were significantly older (all *p* ≤ 0.004), and they had lower PASE scores (all *p* ≤ 0.048). They also had lower mid-upper arm circumference, calf circumference, muscle mass, and bone mass (all *p* < 0.001). In addition, participants with sarcopenia had significantly lower ASMI, handgrip strength, SPPB score, 4-m usual gait speed, and higher 5-time chair test duration (all *p* ≤ 0.038). In terms of nutrition, those with sarcopenia had lower dietary protein intake and poorer nutritional status (MUST) compared to those without sarcopenia (all *p* ≤ 0.015).

**Table 2﻿:** Characteristics of participants by sarcopenic status

	Overall (***n*** = 694)	All participants (***n*** = 694)			Females (***n*** = 429)			Males (***n*** = 265)		
		Non-Sarcopenic (***n*** = 164)	Sarcopenic (***n*** = 530)	***p*** value	Non-Sarcopenic (***n*** = 108)	Sarcopenic (***n*** = 321)	***p*** value	Non-Sarcopenic (***n*** = 56)	Sarcopenic (***n*** = 209)	***p*** value
Age (year)	73.4 ± 0.3	71.5 ± 0.3	74.0 ± 0.3	< 0.001	71.3 ± 0.5	73.5 ± 0.4	0.003	71.9 ± 0.8	74.7 ± 0.5	0.004
Age category, *n* (%)				0.002			0.091			0.008
< 75 years	425 (61)	117 (71)	308 (58)		77 (71)	200 (62)		40 (71)	108 (52)	
≥ 75 years	269 (39)	47 (29)	222 (42)		31 (29)	121 (38)		16 (29)	101 (48)	
MUST score	1.5 ± 0.02	1.3 ± 0.04	1.6 ± 0.03	< 0.001	1.3 ± 0.05	1.6 ± 0.04	< 0.001	1.3 ± 0.08	1.6 ± 0.04	0.007
MUST risk category, *n* (%)				< 0.001			< 0.001			< 0.001
Medium (1)	391 (56)	127 (77)	264 (50)		84 (78)	156 (49)		43 (77)	108 (52)	
High (≥ 2)	303 (44)	37 (23)	266 (50)		24 (22)	165 (51)		13 (23)	101 (48)	
Ethnicity, *n* (%)				0.184			0.602			0.248
Chinese	610 (88)	149 (91)	461 (87)		100 (93)	292 (91)		49 (88)	169 (81)	
Non-Chinese	84 (12)	15 (9)	69 (13)		8 (7)	29 (9)		7 (13)	40 (19)	
Highest level of education, *n* (%)				0.001			< 0.001			0.355
No formal education	243 (35)	47 (29)	196 (37)		30 (28)	122 (38)		17 (30)	74 (35)	
Secondary level or equivalent	284 (41)	59 (36)	225 (43)		38 (35)	141 (44)		21 (38)	84 (40)	
A level or equivalent	108 (16)	39 (24)	69 (13)		30 (28)	35 (11)		9 (16)	34 (16)	
University and above	58 (8)	19 (12)	39 (7)		10 (9)	22 (7)		9 (16)	17 (8)	
Housing type﻿, n (%)				0.106			0.186			0.079
HDB 1–3 room flats	227 (33)	43 (26)	184 (35)		30 (28)	106 (33)		13 (23)	78 (37)	
HDB 4–5 room flats	314 (45)	84 (51)	230 (43)		56 (52)	134 (42)		28 (50)	96 (46)	
Private properties	153 (22)	37 (23)	116 (22)		22 (20)	81 (25)		15 (27)	35 (17)	
Smoking status, *n* (%)				0.082			0.395			0.299
Non-smoker	537 (77)	132 (80)	405 (76)		104 (96)	303 (94)		28 (50)	102 (49)	
Past smoker	99 (14)	25 (15)	74 (14)		3 (3)	7 (2)		22 (39)	67 (32)	
Daily / occasional smoker	58 (8)	7 (4)	51 (10)		1 (1)	11 (3)		6 (11)	40 (19)	
Alcohol consumption, *n* (%)				0.411			0.758			0.471
No alcohol	552 (80)	129 (79)	423 (80)		89 (82)	274 (85)		40 (71)	149 (71)	
< once a month	76 (11)	22 (13)	54 (10)		14 (13)	34 (11)		8 (14)	20 (10)	
≥ once a month	66 (10)	13 (8)	53 (10)		5 (5)	13 (4)		8 (14)	40 (19)	
Physical Activity Scale for the Elderly score	109.8 ± 2.4	123.0 ± 5.1	105.7 ± 2.7	0.002	119.4 ± 5.3	104.0 ± 3.1	0.013	129.8 ± 10.9	108.4 ± 4.7	0.048
Modified Barthel Index score	99.5 ± 0.1	99.4 ± 0.3	99.6 ± 0.1	0.584	99.4 ± 0.4	99.5 ± 0.1	0.718	99.6 ± 0.3	99.7 ± 0.1	0.670
Total Charlson Comorbidity score	0.07 ± 0.01	0.07 ± 0.02	0.07 ± 0.01	0.915	0.04 ± 0.02	0.05 ± 0.01	0.689	0.13 ± 0.05	0.11 ± 0.03	0.723
Number of days admitted to hospital in last 6 months	0.64 ± 0.12	0.42 ± 0.12	0.71 ± 0.16	0.319	0.25 ± 0.13	0.58 ± 0.18	0.289	0.75 ± 0.26	0.89 ± 0.28	0.798
Number of prescribed drugs, *n* (%)				0.135			0.486			0.167
0	195 (28)	56 (34)	139 (26)		35 (32)	87 (27)		21 (38)	52 (25)	
1–5	396 (57)	87 (53)	309 (58)		60 (56)	185 (58)		27 (48)	124 (59)	
≥ 5	103 (15)	21 (13)	82 (15)		13 (12)	49 (15)		8 (14)	33 (16)	
25-hydroxyvitamin D status, *n* (%)				0.027			0.022			0.542
Deficient < 20 μg/L	126 (18)	23 (14)	103 (19)		18 (17)	71 (22)		5 (9)	32 (15)	
Insufficient 20 ﻿– < 30 μg/L	285 (41)	83 (51)	202 (38)		58 (54)	123 (38)		25 (45)	79 (38)	
Sufficient 30 ﻿–100 μg/L	282 (41)	58 (35)	224 (42)		32 (30)	127 (40)		26 (46)	97 (46)	
Possible toxicity > 100 μg/L	1 (0)	0	1 (0)		0	0		0	1 (0)	
25-hydroxyvitamin D (μg/L)	28.7 ± 0.37	28.6 ± 0.71	28.7 ± 0.43	0.906	27.2 ± 0.85	27.6 ± 0.52	0.677	31.3 ± 1.21	30.4 ± 0.75	0.550
Pre-albumin (mg/dL)	24.1 ± 0.17	23.7 ± 0.35	24.2 ± 0.20	0.197	23.8 ± 0.42	23.4 ± 0.23	0.451	23.5 ± 0.63	25.4 ± 0.35	0.011
Creatinine (μmol/L)	73.1 ± 0.84	73.9 ± 1.66	72.8 ± 0.97	0.602	65.6 ± 1.49	62.5 ± 0.80	0.053	89.7 ± 2.94	88.7 ± 1.59	0.774
Height (cm)	156.8 ± 0.33	157.4 ± 0.71	156.6 ± 0.37	0.308	152.8 ± 0.67	152.3 ± 0.36	0.451	166.3 ± 0.75	163.3 ± 0.45	0.002
Body weight (kg)	45.92 ± 0.24	48.84 ± 0.50	45.02 ± 0.26	< 0.001	45.94 ± 0.44	42.54 ± 0.29	< 0.001	54.42 ± 0.77	48.82 ± 0.36	< 0.001
BMI (kg/m^2^)	18.62 ± 0.06	19.66 ± 0.12	18.30 ± 0.07	< 0.001	19.67 ± 0.14	18.32 ± 0.09	< 0.001	19.66 ± 0.21	18.27 ± 0.10	< 0.001
BMI category, *n* (%)				< 0.001			< 0.001			< 0.001
Underweight (< 18.5)	282 (41)	30 (18)	252 (48)		19 (18)	154 (48)		11 (20)	98 (47)	
Normal weight (18.5–24.9)	411 (59)	133 (81)	278 (52)		88 (81)	167 (52)		45 (80)	111 (53)	
Overweight (25–29.9)	1 (0)	1 (< 1)	0		1 (1)	0		0	0	
Obese (≥ 30.0)	0	0	0		0	0		0	0	
Mid-upper arm circumference (cm)	23.04 ± 0.09	24.10 ± 0.19	22.72 ± 0.10	< 0.001	23.61 ± 0.21	22.28 ± 0.13	< 0.001	25.05 ± 0.35	23.38 ± 0.15	< 0.001
Calf circumference (cm)	30.81 ± 0.10	32.45 ± 0.19	30.31 ± 0.11	< 0.001	31.94 ± 0.21	29.68 ± 0.14	< 0.001	33.44 ± 0.34	31.28 ± 0.15	< 0.001
Fat mass (kg)	8.54 ± 0.13	8.82 ± 0.28	8.45 ± 0.14	0.220	10.09 ± 0.31	9.70 ± 0.17	0.261	6.37 ± 0.40	6.54 ± 0.17	0.669
Muscle mass (kg)	35.40 ± 0.23	37.86 ± 0.52	34.64 ± 0.25	< 0.001	33.88 ± 0.31	31.12 ± 0.18	< 0.001	45.54 ± 0.56	40.05 ± 0.29	< 0.001
Bone mass (kg)	1.98 ± 0.01	2.16 ± 0.03	1.92 ± 0.02	< 0.001	1.97 ± 0.02	1.72 ± 0.02	< 0.001	2.51 ± 0.03	2.23 ± 0.02	< 0.001
Dietary protein intake (g/d)	52.2 ± 0.54	55.6 ± 1.13	51.1 ± 0.61	< 0.001	53.3 ± 1.43	49.5 ± 0.78	0.015	59.9 ± 1.71	53.7 ± 0.94	0.002
Total energy intake (kcal/d)	1112 ± 10.8	1168 ± 22.1	1094 ± 12.2	0.004	1098 ± 27.1	1052 ± 15.6	0.142	1303 ± 31.3	1159 ± 18.9	< 0.001
Energy-adjusted protein intake (g/d)	52.2 ± 0.4	52.9 ± 0.4	52.1 ± 0.2	0.047	52.7 ± 0.5	51.8 ± 0.3	0.094	53.3 ± 0.7	52.5 ± 0.3	0.237
ASMI (kg/m^2^)	5.73 ± 0.03	6.34 ± 0.06	5.55 ± 0.03	< 0.001	5.91 ± 0.04	5.13 ± 0.02	< 0.001	7.17 ± 0.07	6.19 ± 0.04	< 0.001
Handgrip strength (kg)	17.4 ± 0.2	18.9 ± 0.6	16.9 ± 0.2	< 0.001	15.6 ± 0.4	13.9 ± 0.2	< 0.001	25.2 ± 0.9	21.5 ± 0.4	< 0.001
Handgrip endurance (s)	71.5 ± 2.0	80.2 ± 5.1	68.8 ± 2.1	0.015	77.9 ± 6.6	68.3 ± 2.7	0.111	84.4 ± 7.6	69.7 ± 3.2	0.044
Leg strength (kg)	12.0 ± 0.2	12.9 ± 0.4	11.8 ± 0.2	0.007	11.8 ± 0.4	11.0 ± 0.2	0.089	14.9 ± 0.7	12.9 ± 0.3	0.005
SPPB score	9.2 ± 0.1	9.8 ± 0.2	9.1 ± 0.1	< 0.001	9.7 ± 0.2	9.1 ± 0.1	0.005	9.9 ± 0.2	9.1 ± 0.1	0.002
4-m usual gait speed (m/s)	0.87 ± 0.01	0.94 ± 0.02	0.85 ± 0.01	< 0.001	0.93 ± 0.03	0.84 ± 0.01	0.001	0.95 ± 0.04	0.86 ± 0.02	0.010
5-time chair stand test duration (s)	15.7 ± 0.2	14.7 ± 0.41	16.1 ± 0.23	0.003	14.8 ± 0.5	15.7 ± 0.3	0.038	14.5 ± 0.6	16.2 ± 0.4	0.029

Note: For continuous variables, mean ± standard error is presented; for categorical variables, n (%) is presented. The sample sizes for some variables are less than the overall stated sample sizes

Table 3 shows the factors associated with sarcopenia using univariate logistic regression in the overall cohort, in females, and in males. These factors included age, MUST risk, BMI, mid-upper arm circumference, calf circumference, bone mass, total SPPB score, and 5-time chair stand test score (all *p* ≤ 0.028).

**Table 3:** Factors associated with sarcopenia using univariate logistic regression

	Overall cohort			Females			Males		
	Odds ratio	95% CI	***p*** value	Odds ratio	95% CI	***p*** value	Odds ratio	95% CI	***p*** value
Gender			0.224						
Male (ref)	1.00								
Female	0.80	0.55, 1.15							
Age (year)	1.07	1.03, 1.10	< 0.001	1.06	1.02, 1.10	0.003	1.08	1.02, 1.13	0.005
Age			0.003			0.092			0.009
< 75 years (ref)	1.00			1.00			1.00		
≥ 75 years	1.79	1.23, 2.62		1.50	0.94, 2.41		2.34	1.23, 4.43	
MUST Score	2.54	1.75, 3.70	< 0.001	2.76	1.72, 4.44	< 0.001	2.25	1.23, 4.14	0.009
MUST risk			< 0.001			< 0.001			0.001
Medium (ref)	1.00			1.00			1.00		
High	3.46	2.31, 5.18		3.70	2.24, 6.13		3.09	1.57, 6.09	
Ethnicity			0.187			0.603			0.252
Chinese (ref)	1.00			1.00			1.00		
Non-Chinese	1.49	0.83, 2.68		1.24	0.55, 2.80		1.66	0.70, 3.93	
Education			0.002			< 0.001			0.372
No formal education (ref)	1.00			1.00			1.00		
Secondary level or equivalent	0.91	0.60, 1.40	0.683	0.91	0.53, 1.56	0.738	0.92	0.45, 1.87	0.816
A level or equivalent	0.42	0.26, 0.70	< 0.001	0.29	0.15, 0.54	< 0.001	0.87	0.35, 2.14	0.759
University and above	0.49	0.26, 0.93	0.028	0.54	0.23, 1.26	0.155	0.43	0.17, 1.14	0.090
Housing type			0.108			0.188			0.085
HDB 1–3 room flats (ref)	1.00			1.00			1.00		
HDB 4–5 room flats	0.64	0.42, 0.97	0.035	0.68	0.41, 1.13	0.135	0.57	0.28, 1.18	0.129
Private properties	0.73	0.45, 1.20	0.220	1.04	0.56, 1.94	0.897	0.39	0.17, 0.90	0.028
Smoking status			0.108			0.425			0.296
Non-smoker (ref)	1.00			1.00			1.00		
Past smoker	0.96	0.59, 1.58	0.887	0.80	0.20, 3.15	0.751	0.84	0.44, 1.58	0.582
Daily / occasional smoker	2.37	1.05, 5.36	0.037	3.77	0.48, 29.52	0.206	1.83	0.70, 4.75	0.215
Alcohol consumption			0.414			0.759			0.476
None (ref)	1.00			1.00			1.00		
Less than once a month	0.75	0.44, 1.28	0.287	0.79	0.40, 1.54	0.486	0.67	0.28, 1.64	0.380
≥ once a month	1.24	0.66, 2.35	0.504	0.84	0.29, 2.43	0.754	1.34	0.58, 3.09	0.490
Number of prescribed drugs currently taking			0.137			0.488			0.172
0 (ref)	1.00			1.00			1.00		
1–5	1.43	0.97, 2.12	0.072	1.24	0.76, 2.02	0.387	1.85	0.96, 3.57	0.065
> 5	1.57	0.89, 2.78	0.120	1.52	0.73, 3.14	0.261	1.67	0.66, 4.20	0.279
Modified Barthel Index score	1.02	0.95, 1.09	0.586	1.01	0.94, 1.09	0.719	1.03	0.89, 1.21	0.671
Physical Activity Scale for the Elderly score	1.00	0.99, 1.00	0.002	1.00	0.99, 1.00	0.014	1.00	0.99, 1.00	0.051
Total Charlson Comorbidity score	1.03	0.56, 1.92	0.916	1.25	0.43, 3.65	0.689	0.87	0.41, 1.86	0.722
Body weight (kg)	0.91	0.88, 0.93	< 0.001	0.87	0.83, 0.92	< 0.001	0.81	0.75,0 .87	< 0.001
BMI (kg/m^2^)	0.53	0.45, 0.61	< 0.001	0.55	0.46, 0.66	< 0.001	0.48	0.37, 0.63	< 0.001
BMI			< 0.001			< 0.001			< 0.001
< 18.5 kg/m2 ﻿(ref)	1.00			1.00			1.00		
18.5–24.9 kg/m2﻿	0.25	0.16, 0.38	< 0.001	0.23	0.14, 0.40	< 0.001	0.28	0.14, 0.56	< 0.001
Fat mass (kg)	0.97	0.92, 1.02	0.220	0.96	0.90, 1.03	0.261	1.03	0.91, 1.15	0.667
Bone mass (100 g)	0.83	0.79, 0.88	< 0.001	0.73	0.66, 0.80	< 0.001	0.52	0.43, 0.63	< 0.001
Mid upper arm circumference (cm)	0.78	0.72, 0.84	< 0.001	0.77	0.69, 0.85	< 0.001	0.73	0.64, 0.84	< 0.001
Calf circumference (cm)	0.68	0.62, 0.74	< 0.001	0.65	0.58, 0.73	< 0.001	0.63	0.53, 0.75	< 0.001
25-hydroxyvitamin D (μg/L)	1.00	0.98, 1.02	0.906	1.00	0.98, 1.03	0.676	0.99	0.97, 1.02	0.549
25-hydroxyvitamin D status			0.017			0.021			0.413
Sufficient, 30 –﻿ < 100 μg/L (ref)	1.00			1.00			1.00		
Insufficient, 20 –﻿ < 30 μg/L	0.63	0.43, 0.93	0.019	0.53	0.32, 0.88	0.014	0.85	0.45, 1.58	0.602
Deficient, < 20 μg/L	1.16	0.68, 1.98	0.589	0.99	0.52, 1.90	0.985	1.72	0.61, 4.84	0.308
Pre-albumin (mg/dL)	1.03	0.99, 1.07	0.197	0.98	0.93, 1.03	0.451	1.08	1.02, 1.15	0.013
Creatinine (μmol/L)	1.00	0.99, 1.01	0.601	0.99	0.97, 1.00	0.056	1.00	0.99, 1.01	0.773
Dietary protein intake (g/d)	0.98	0.97, 0.99	< 0.001	0.98	0.97, 1.00	0.016	0.97	0.95, 0.99	0.003
Total energy intake (kcal/d)	1.00	1.00, 1.00	0.004	1.00	1.00, 1.00	0.143	1.00	1.00, 1.00	< 0.001
Energy-adjusted protein intake (g/d)	0.98	0.97, 1.00	0.041	0.98	0.96, 1.00	0.052	0.99	0.96, 1.02	0.414
Leg strength (kg)	0.95	0.91, 0.99	0.007	0.96	0.91, 1.01	0.090	0.92	0.86, 0.98	0.007
Handgrip endurance (s)	1.00	0.99, 1.00	0.017	1.00	0.99, 1.00	0.114	0.99	0.99, 1.00	0.047
Total SPPB score	0.81	0.73, 0.90	< 0.001	0.84	0.75, 0.95	0.005	0.74	0.61, 0.90	0.002
Total balance test score	0.71	0.52, 0.97	0.034	0.79	0.56, 1.11	0.176	0.43	0.18, 1.01	0.053
﻿5-time chair stand test score	0.72	0.61, 0.85	< 0.001	0.77	0.63, 0.94	0.011	0.65	0.48, 0.86	0.003
Gait speed test score	0.74	0.59, 0.92	0.007	0.74	0.56, 0.97	0.028	0.72	0.49, 1.08	0.114

Table 4 shows the results from multiple logistic regression models for overall cohort, females, and males. In the overall cohort, after adjusting for confounders, sarcopenia was associated with older age, male gender, and higher risk of malnutrition. On the other hand, greater calf circumference and bone mass were associated with lower odds of having sarcopenia (Table 4) (all *p* ≤ 0.044). For every one-year increase in age holding other factors constant, the odds of having sarcopenia was 1.04 (95% CI: 1.00, 1.08). Females had significantly lower odds of having sarcopenia compared to males (0.15, 95% CI: 0.07, 0.29). Compared to older adults at medium risk of malnutrition, the odds of having sarcopenia was significantly higher among those at high risk of malnutrition (2.11, 95% CI: 1.32, 3.36). Calf circumference and bone mass were associated with lower odds of having sarcopenia (both *p* < 0.001).

**Table 4:** Factors associated with sarcopenia using multiple logistic regression models

	Overall cohort			Females			Males		
	Odds Ratio	95% CI	***p*** value	Odds Ratio	95% CI	***p*** value	Odds Ratio	95% CI	***p*** value
Gender			< 0.001						
Male	1.00								
Female	0.15	0.07, 0.29	< 0.001						
Age (year)	1.04	1.00, 1.08	0.044	1.03	0.98, 1.08	0.225	1.02	0.96, 1.10	0.476
Ethnicity			0.829			0.346			0.633
Chinese	1.00			1.00			1.00		
Non-Chinese	0.92	0.45, 1.90	0.829	0.63	0.24, 1.66	0.346	1.34	0.41, 4.38	0.633
MUST risk			0.002			0.001			0.576
Medium	1.00			1.00			1.00		
High	2.11	1.32, 3.36	0.002	2.61	1.46, 4.67	0.001	1.27	0.55, 2.96	0.576
Calf circumference (cm)	0.78	0.70, 0.87	< 0.001	0.77	0.67, 0.88	< 0.001	0.79	0.63, 0.98	0.033
Bone mass (100 g)	0.77	0.70, 0.85	< 0.001	0.82	0.73, 0.92	< 0.001	0.58	0.46, 0.73	< 0.001
Physical Activity Scale for the Elderly score	1.00	0.99, 1.00	0.145	1.00	0.99, 1.00	0.094	1.00	0.99, 1.00	0.354
Education			0.075			0.003			0.624
No formal education	1.00			1.00			1.00		
Secondary level or equivalent	1.22	0.72, 2.05	0.462	1.18	0.62, 2.26	0.615	1.05	0.41, 2.71	0.922
A level or equivalent	0.57	0.31, 1.04	0.065	0.33	0.15, 0.70	0.004	2.09	0.65, 6.80	0.218
University and above	1.02	0.48, 2.20	0.952	1.04	0.37, 2.96	0.936	1.12	0.32, 3.86	0.862
Smoking status			0.168			0.653			0.227
Non-smoker	1.00			1.00			1.00		
Past smoker	0.63	0.31, 1.26	0.190	0.95	0.17, 5.22	0.949	0.49	0.21, 1.16	0.107
Daily / occasional smoker	1.76	0.63, 4.96	0.282	3.44	0.25, 47.85	0.357	1.10	0.32, 3.84	0.878
Alcohol consumption			0.293			0.599			0.415
None	1.00			1.00			1.00		
< once a month	1.44	0.73, 2.83	0.297	1.53	0.67, 3.50	0.313	1.31	0.37, 4.70	0.678
≥ once a month	1.69	0.77, 3.70	0.188	1.12	0.31, 4.07	0.858	2.05	0.70, 6.00	0.188

## Discussion

To our knowledge, this is the first large-scale study of its kind that has reported the prevalence of sarcopenia and examined a wide range of associated factors in community-dwelling, relatively well, and independently living older adults who were at risk of malnutrition in Singapore. Given the high likelihood of both malnutrition and sarcopenia in community-dwelling older adults, we sought to quantify the clinical relationship between the two.

Results of our study highlight the high prevalence of sarcopenia in older adults who were at risk of malnutrition, i.e., 76% in the overall population with 57% qualifying as severe sarcopenia. Compared to participants without sarcopenia, those with sarcopenia were older, had lower dietary protein intake and PASE score. After adjusting for confounders, sarcopenia was significantly associated with older age, male gender, higher risk of malnutrition, lower calf circumference, and lower bone mass. As malnutrition risk (based on MUST risk category) increased from medium to high, risk for sarcopenia doubled. These findings suggest there is a need to conduct concomitant screening for muscle health and nutritional health in at-risk older adults. Early interventions can then be instituted to prevent further progression and to reverse deficits for both muscle and nutritional health [2, 41–43].

In the present study, using the individual criteria of the AWGS updated consensus 2019 [2], 81% of participants had low ASMI, 83% had low muscle strength, and 78% had low physical performance. Across Asia, low muscle mass was reported to occur commonly in community-dwelling older adults, ranging from 20 to 65% [12, 44–47]. Specifically referring to sarcopenia in older community-living Asian adults, the prevalence ranged from 18 to 46% [3, 8–12]. The prevalence range is also widely varied in countries around the world [48]; prevalence varies according to age [49] and community-dwelling or site of care [50, 51]. In a large meta-analysis of research studies around the world, the prevalence of sarcopenia in older adults varied between 10 and 27% for those ≥ 60 years; in this analysis, the highest prevalence rates were in Europe, while lower rates were found for Oceania and Asia [48]. Another meta-analysis of studies in multiple countries showed sarcopenia prevalence range of 1 to 29% in community-dwelling older adults and 14 to 33% in long-term care [50]. Most results were in the range of 20 to 40%, with the highest prevalence of 68% in the very old (≥ 80 years) [9]. In the present study, we found a sarcopenia prevalence of 76% because we focused on a population that was at risk of malnutrition.

There are differences in cut-off values and in the algorithm used to define sarcopenia when comparing the different versions of each guideline, e.g., AWGS 2019 [2] versus AWGS 2014 [52], and between the Asian versus the European guidelines [2, 53]. For example, the handgrip strength for men has increased from 26 kg to 28 kg, and the gait speed has increased from 0.8 m/s to 1.0 m/s in the AWGS 2019 guidelines. Five-time chair stand test is used as a surrogate marker for gait speed [37]. In terms of the differences between Asian and European guidelines, the Asian guideline includes both strength or physical performance as part of the diagnostic criteria for sarcopenia [2] whereas for the European guideline, physical performance is used as a measure of severity of sarcopenia [53]. For the results of the diagnostic algorithm to be meaningful, population specific cut-offs are essential. Hence, we have applied AWGS cut-offs for our study. For our study cohort, the prevalence of sarcopenia as determined by the AWGS 2019 was 76% versus 70% using AWGS 2014. The higher prevalence is due to the higher cut-offs in handgrip strength and gait speed in AWGS 2019 versus 2014. Two recent studies also reported this observation [12, 54].

Our findings confirm and extend reports from other Asian studies with regards the close association between malnutrition and sarcopenia. Malnutrition is an important contributor to poor muscle health due to many factors. In an energy deficient diet, amino acids and muscle are broken down and oxidized to generate energy to sustain life preserving functions. In a protein deficient diet, muscle protein turnover is in favor of muscle protein breakdown leading to a negative nitrogen balance and progressive loss of both muscle mass and function [42, 55]. Malnutrition also leads to deficiencies of micronutrients that are essential for the integrity and function of muscle, e.g., vitamin D and vitamin B12, leading to further muscle loss and function [56, 57]. This state of low energy, low muscle mass and low muscle function results in low physical strength and power, low physical activity, and low exercise tolerance. This low physical activity and low resting metabolic rate state then contributes to further reduced appetite and malnutrition, thereby completing the vicious perpetual cycle of malnutrition, sarcopenia, and frailty [58–61]. Hence in the early stages of this vicious cycle, malnutrition can be seen as the preceding condition which ultimately leads to sarcopenia and frailty.

In addition to the significant increase in risk of sarcopenia in malnourished versus normal nutrition in older adults, with odds ratio ranging from 9.9 to 13.6 [11, 29], our findings show that there is a doubling of odds ratio for sarcopenia amongst those at high risk versus medium risk of malnutrition. This association is further highlighted in a recent study from our group, which reported that compared to older adults with normal nutritional status, the odds of having low ASMI was 3.58 for those at medium risk of malnutrition and 12.50 for those at high risk of malnutrition [45]. Taken together, the presence of malnutrition is a strong predictor of concomitant poor muscle health. Hence, preventing and addressing malnutrition in older adults is of utmost importance to ensure good muscle health.

In the AWGS 2019 consensus guidelines, sarcopenia is considered an age-related condition of muscle loss and impairment [2]. In a community-dwelling Singaporean adult population (21 to 90 years old, *n* = 541), more than a third of participants (35%) who were at nutritional risk (determined by Mini Nutritional Assessment Short Form, MNA-SF) had sarcopenia [62]. In our study, the prevalence of sarcopenia was more than twice as high due to our cohort being adults aged 65 years and above only.

This age-related increase in sarcopenia prevalence has been widely reported [8, 13, 14]. For example, Pang et al. studied a Singaporean community population of adults and found 13.6% prevalence of sarcopenia in the full population (21 to 90 years old) but the prevalence was 32% among those older than 60 years [12]. Similarly, in a Vietnamese community study where participants had a higher mean age of 70 years, the overall prevalence of sarcopenia was also higher at 55% and older age was associated with a higher risk for sarcopenia [20]. The highest prevalence for sarcopenia of 68% was found in the very old (≥ 80 years) in a study from Thailand [9]. These findings in the literature highlight the important relationship between aging and sarcopenia.

Results of our current study showed that risk for sarcopenia was significantly greater for men than for women. Although the literature regarding gender-related differences shows inconsistency [63], our findings were similar to other Asian studies which had also found a higher prevalence in men [12, 14, 20, 64]. Thus, further research is required in this area.

Calf circumference is a recognized surrogate marker for muscle mass [53] and is also used as one of the criteria for case finding in sarcopenia [2]. A recent study from our group using the receiver operating characteristic (ROC) curve analysis reported that the cut-off values of calf circumference for low ASMI for men was < 33.4 cm and for women was < 32.2 cm [45], which is in line with AWGS 2019 consensus update case finding for sarcopenia cut-offs set at < 34 cm for men and < 33 cm for women [2]. Previous studies have reported that higher calf circumference was associated with lower odds of having sarcopenia [14, 15], and this relationship was also observed in the present study.

There is a known relationship between muscle health and bone health. Our study found an inverse association between sarcopenia and bone mass. Previous studies have reported that low bone mass was associated with sarcopenia [16, 17], with the prevalence of osteoporosis increased with increasing severity of sarcopenia [65]. Interventions that improve muscle health are likely to improve bone health [66]. Hence, older adults with low bone mineral density should also be screened for sarcopenia so that interventions can be initiated where appropriate to improve both muscle and bone health.

Research groups have also reported associations between sarcopenia and the presence of co-morbid disease conditions such as diabetes [18, 19] and cognitive impairment [21]. Nguyen et al. [20] found that malnutrition and chronic lung disease were associated with sarcopenia with odds ratio of 3.77 and 3.48 respectively. Our study population included community-dwelling older adults at risk of malnutrition who were otherwise relatively healthy, as determined by the Charlson comorbidity score. As such, the prevalence could be even higher in older adults with malnutrition risk and the additional burden of comorbidities.

Our study showed that compared to participants without sarcopenia, those with sarcopenia had lower dietary protein intake and had a lower mean PASE score. This relationship between sarcopenia and physical activity was highlighted in a recent study where moderate and low physical activity levels were associated with higher odds of having sarcopenia (OR of 4.12 and 7.02 respectively) compared to high physical activity levels [20].

Protein intake and physical activity are modifiable risk factors for sarcopenia, so these are ideal targets to prevent or delay the onset of sarcopenia [41, 42, 67–71]. Chew and others recently summarized evidence-based use of nutrition and progressive resistance exercise training as interventions to improve muscle health in older Asian adults [30]. In addition, our group recently reported that specialized oral nutritional supplement with dietary counseling significantly improved nutritional and functional outcomes in community-dwelling older adults at risk of malnutrition [31].

There is a strong and consistent finding from our study and others, which highlights that community screening of older adults for characteristics that reflect both nutritional status and muscle health could facilitate the early initiation of appropriate interventions to mitigate risk of adverse health outcomes for both malnutrition and sarcopenia before they become established.

Importantly, our findings revealed that among community-dwelling older adults at risk of malnutrition, over three quarters had sarcopenia. We found that the greater the malnutrition severity, the higher the risk for sarcopenia. Thus, screening, diagnosing, and treating malnutrition in community-dwelling older adults could be a useful strategy for concomitant early detection and management of sarcopenia in the same individuals. Validated and easy-to-use screening tools such as MUST and Mini Nutritional Assessment Short Form (MNA-SF) can be used to determine risk of malnutrition in older populations [72].

Our study was limited in that it was a cross-sectional design. We are thus constrained to report an association between malnutrition and sarcopenia, but we cannot prove that malnutrition was causal. We used BIA as a measure of muscle mass in our study. BIA has been validated [73, 74] and is recognized as one of the methods to measure muscle mass by AWGS [2] as well as by EWGSOP [53]. It is easy to use, inexpensive, safe, and non-invasive. However, it is an indirect measure of muscle mass, which is affected by the physical condition of the participants, such as hydration and extreme body mass index [75]. Such effects may influence the determination of low muscle mass when compared to use of whole body DEXA for the same cohort. Nevertheless, BIA is commonly used to determine the prevalence of low muscle mass and sarcopenia [47, 76, 77]. The other limitation of our study is also its’ strength, as our study cohort included relatively well, independently living older adults (as defined by the study eligibility criteria) hence limiting the generalizability of our findings. Importantly, it appears that the risk of being malnourished and sarcopenic will be even higher and more severe in cohorts of older adults with multimorbidity, inactivity, and frailty.

## Conclusions

Sarcopenia and malnutrition are prevalent among older people and often co-exist in the same individual. In the present study, we found that three out of every four community-dwelling older adults at risk of malnutrition also fulfilled criteria for the diagnosis of sarcopenia. This suggests that the benefits of routine screening for malnutrition risk in community-dwelling older adults extends beyond nutrition and can facilitate the early diagnosis and management of sarcopenia. Addressing both malnutrition and sarcopenia is key to healthy aging and to supporting independent living for as long as possible in later life. Evidence-based interventions such as oral nutritional supplements and resistance exercise training are available and imperative.

## Data Availability

The datasets used and/or analyzed during the current study available from the corresponding author on reasonable request.

## Abbreviations

ASMI: Appendicular skeletal muscle mass index
ASMM: Appendicular skeletal muscle mass
AWGS: Asian Working Group for Sarcopenia
BIA: Bioelectrical impedance analysis
BMI: Body mass index
CCI: Charlson Comorbidity Index
CI: Confidence interval
COPD: Chronic obstructive pulmonary disease
MBI: Modified Barthel Index
MNA-SF: Mini Nutritional Assessment Short Form
MUAC: Mid-upper arm circumference
MUST: Malnutrition Universal Screening Tool
OR: Odds ratio
PASE: Physical Activity Scale for the Elderly
ROC: Receiver operating characteristic
SHIELD: Strengthening Health In ELDerly through nutrition
SPPB: Short Physical Performance Battery

## References

[1] United Nations (2019). World population ageing 2019: highlights.

[2] Chen L-K, Woo J, Assantachai P, Auyeung T-W, Chou M-Y, Iijima K (2020). Asian working Group for Sarcopenia: 2019 consensus update on sarcopenia diagnosis and treatment. J Am Med Dir Assoc.

[3] Chang HK, Lee JY, Gil CR, Kim MK (2020). Prevalence of sarcopenia in community-dwelling older adults according to simplified algorithms for sarcopenia consensus based on Asian working Group for Sarcopenia. Clin Interv Aging.

[4] Gao L, Jiang J, Yang M, Hao Q, Luo L, Dong B (2015). Prevalence of sarcopenia and associated factors in Chinese community-dwelling elderly: comparison between rural and urban areas. J Am Med Dir Assoc.

[5] Shafiee G, Keshtkar A, Soltani A, Ahadi Z, Larijani B, Heshmat R (2017). Prevalence of sarcopenia in the world: a systematic review and meta- analysis of general population studies. J Diabetes Metab Disord.

[6] Shiota A, Nakayama N, Saito Y, Maeda T, Maeda Y, Nakayama K (2020). Prevalence and associated factors of malnutrition and sarcopenia in a daycare facility: a cross-sectional study. Healthcare (Basel).

[7] Yap SF, Boo NY, Pramod DS, Thaw Z, Liew SF, Woo LF (2020). Risk factors associated with sarcopenia among independently mobile, institutionalised older people in the Klang valley of Malaysia: a cross-sectional study. Malays J Med Sci.

[8] Chen X, Hou L, Zhang Y, Dong B (2021). Analysis of the prevalence of sarcopenia and its risk factors in the elderly in the Chengdu community. J Nutr Health Aging.

[9] Khongsri N, Tongsuntud S, Limampai P, Kuptniratsaikul V (2016). The prevalence of sarcopenia and related factors in a community-dwelling elders Thai population. Osteoporos Sarcopenia.

[10] Keng BMH, Gao F, Teo LLY, Lim WS, Tan RS, Ruan W (2019). Associations between skeletal muscle and myocardium in aging: a syndrome of “cardio-sarcopenia”?. J Am Geriatr Soc.

[11] Lu Y, Karagounis LG, Ng TP, Carre C, Narang V, Wong G (2020). Systemic and metabolic signature of sarcopenia in community-dwelling older adults. J Gerontol A Biol Sci Med Sci.

[12] Pang BWJ, Wee S-L, Lau LK, Jabbar KA, Seah WT, Ng DHM (2021). Prevalence and associated factors of sarcopenia in Singaporean adults - the Yishun study. J Am Med Dir Assoc.

[13] Therakomen V, Petchlorlian A, Lakananurak N (2020). Prevalence and risk factors of primary sarcopenia in community-dwelling outpatient elderly: a cross-sectional study. Sci Rep.

[14] Nasimi N, Dabbaghmanesh MH, Sohrabi Z (2019). Nutritional status and body fat mass: determinants of sarcopenia in community-dwelling older adults. Exp Gerontol.

[15] Kim H, Suzuki T, Kim M, Kojima N, Yoshida Y, Hirano H (2015). Incidence and predictors of sarcopenia onset in community-dwelling elderly Japanese women: 4-year follow-up study. J Am Med Dir Assoc.

[16] Cheng Q, Zhu X, Zhang X, Li H, Du Y, Hong W (2014). A cross-sectional study of loss of muscle mass corresponding to sarcopenia in healthy Chinese men and women: reference values, prevalence, and association with bone mass. J Bone Miner Metab.

[17] Intriago M, Maldonado G, Guerrero R, Messina OD, Rios C (2020). Bone mass loss and sarcopenia in Ecuadorian patients. Journal of Aging Research.

[18] Chung SM, Moon JS, Chang MC (2021). Prevalence of sarcopenia and its association with diabetes: a meta-analysis of community-dwelling Asian population. Front Med (Lausanne).

[19] Han P, Kang L, Guo Q, Wang J, Zhang W, Shen S (2016). Prevalence and factors associated with sarcopenia in suburb-dwelling older Chinese using the Asian working Group for Sarcopenia definition. J Gerontol A Biol Sci Med Sci.

[20] Nguyen TN, Nguyen TN, Nguyen AT, Nguyen TX, Nguyen HTT, Nguyen TTH (2020). Prevalence of sarcopenia and its associated factors in patients attending geriatric clinics in Vietnam: a cross-sectional study. BMJ Open.

[21] Liu X, Hou L, Xia X, Liu Y, Zuo Z, Zhang Y (2020). Prevalence of sarcopenia in multi ethnics adults and the association with cognitive impairment: findings from West-China health and aging trend study. BMC Geriatr.

[22] Wei K, Nyunt MS, Gao Q, Wee SL, Yap KB, Ng TP (2018). Association of frailty and malnutrition with long-term functional and mortality outcomes among community-dwelling older adults: results from the Singapore longitudinal aging study 1. JAMA Netw Open.

[23] Trevisan C, Crippa A, Ek S, Welmer AK, Sergi G, Maggi S (2019). Nutritional status, body mass index, and the risk of falls in community-dwelling older adults: a systematic review and meta-analysis. J Am Med Dir Assoc.

[24] Wei K, Nyunt MSZ, Gao Q, Wee SL, Ng TP (2019). Long-term changes in nutritional status are associated with functional and mortality outcomes among community-living older adults. Nutrition..

[25] Agarwal E, Miller M, Yaxley A, Isenring E (2013). Malnutrition in the elderly: a narrative review. Maturitas..

[26] Soderstrom L, Rosenblad A, Thors Adolfsson E, Bergkvist L (2017). Malnutrition is associated with increased mortality in older adults regardless of the cause of death. Br J Nutr.

[27] Yeung SSY, Chan RSM, Kwok T, Lee JSW, Woo J (2021). Malnutrition according to GLIM criteria and adverse outcomes in community-dwelling Chinese older adults: a prospective analysis. J Am Med Dir Assoc.

[28] Beaudart C, Sanchez-Rodriguez D, Locquet M, Reginster J-Y, Lengelé L, Bruyère O (2019). Malnutrition as a strong predictor of the onset of sarcopenia. Nutrients..

[29] Sato PHR, Ferreira AA, Rosado EL (2020). The prevalence and risk factors for sarcopenia in older adults and long-living older adults. Arch Gerontol Geriatr.

[30] Chew STH, Kayambu G, Lew CCH, Ng TP, Ong F, Tan J (2021). Singapore multidisciplinary consensus recommendations on muscle health in older adults: assessment and multimodal targeted intervention across the continuum of care. BMC Geriatr.

[31] Chew STH, Tan NC, Cheong M, Oliver J, Baggs G, Choe Y (2021). Impact of specialized oral nutritional supplement on clinical, nutritional, and functional outcomes: a randomized, placebo-controlled trial in community-dwelling older adults at risk of malnutrition. Clin Nutr.

[32] Stratton RJ, Hackston A, Longmore D, Dixon R, Price S, Stroud M (2004). Malnutrition in hospital outpatients and inpatients: prevalence, concurrent validity and ease of use of the 'Malnutrition universal screening Tool' ('MUST') for adults. Br J Nutr.

[33] Charlson ME, Pompei P, Ales KL, MacKenzie CR (1987). A new method of classifying prognostic comorbidity in longitudinal studies: development and validation. J Chronic Dis.

[34] Washburn RA, Smith KW, Jette AM, Janney CA (1993). The physical activity scale for the elderly (PASE): development and evaluation. J Clin Epidemiol.

[35] Shah S, Vanclay F, Cooper B (1989). Improving the sensitivity of the Barthel index for stroke rehabilitation. J Clin Epidemiol.

[36] Guralnik JM, Simonsick EM, Ferrucci L, Glynn RJ, Berkman LF, Blazer DG (1994). A short physical performance battery assessing lower extremity function: association with self-reported disability and prediction of mortality and nursing home admission. J Gerontol.

[37] Nishimura T, Arima K, Okabe T, Mizukami S, Tomita Y, Kanagae M (2017). Usefulness of chair stand time as a surrogate of gait speed in diagnosing sarcopenia. Geriatr Gerontol Int.

[38] Kirk B, Zanker J, Bani Hassan E, Bird S, Brennan-Olsen S, Duque G (2021). Sarcopenia definitions and outcomes consortium (SDOC) criteria are strongly associated with malnutrition, depression, falls, and fractures in high-risk older persons. J Am Med Dir Assoc.

[39] Kitamura A, Seino S, Abe T, Nofuji Y, Yokoyama Y, Amano H (2021). Sarcopenia: prevalence, associated factors, and the risk of mortality and disability in Japanese older adults. J Cachexia Sarcopenia Muscle.

[40] Mo YH, Zhong J, Dong X, Su YD, Deng WY, Yao XM (2021). Comparison of three screening methods for sarcopenia in community-dwelling older persons. J Am Med Dir Assoc.

[41] Chen L-K, Arai H, Assantachai P, Akishita M, Chew STH, Dumlao LC (2022). Roles of nutrition in muscle health of community-dwelling older adults: evidence-based expert consensus from Asian working Group for Sarcopenia. J Cachexia Sarcopenia Muscle.

[42] Prado CM, Landi F, Chew STH, Atherton PJ, Molinger J, Ruck T (2022). Advances in muscle health and nutrition: a toolkit for healthcare professionals. Clin Nutr.

[43] Volkert D, Beck AM, Cederholm T, Cruz-Jentoft A, Hooper L, Kiesswetter E (2022). ESPEN practical guideline: clinical nutrition and hydration in geriatrics. Clin Nutr.

[44] Kim M, Kim J, Won CW (2018). Association between involuntary weight loss with low muscle mass and health-related quality of life in community-dwelling older adults: Nationwide surveys (KNHANES 2008-2011). Exp Gerontol.

[45] Tey SL, Huynh DTT, Berde Y, Baggs G, How CH, Low YL (2021). Prevalence of low muscle mass and associated factors in community-dwelling older adults in Singapore. Sci Rep.

[46] Xu H-q, Shi J-p, Shen C, Liu Y, Liu J-M, Zheng X-y (2019). Sarcopenia-related features and factors associated with low muscle mass, weak muscle strength, and reduced function in Chinese rural residents: a cross-sectional study. Arch Osteoporos.

[47] Zhang Y, Chen X, Hou L, Lin X, Qin D, Wang H (2020). Prevalence and risk factors governing the loss of muscle function in elderly sarcopenia patients: a longitudinal study in China with 4 years of follow-up. J Nutr Health Aging.

[48] Petermann-Rocha F, Balntzi V, Gray SR, Lara J, Ho FK, Pell JP (2022). Global prevalence of sarcopenia and severe sarcopenia: a systematic review and meta-analysis. J Cachexia Sarcopenia Muscle.

[49] von Haehling S, Morley JE, Anker SD (2010). An overview of sarcopenia: facts and numbers on prevalence and clinical impact. J Cachexia Sarcopenia Muscle.

[50] Cruz-Jentoft AJ, Landi F, Schneider SM, Zuniga C, Arai H, Boirie Y (2014). Prevalence of and interventions for sarcopenia in ageing adults: a systematic review. Report of the international sarcopenia initiative (EWGSOP and IWGS). Age Ageing.

[51] Darroch P, O’Brien WJ, Mazahery H, Wham C (2022). Sarcopenia prevalence and risk factors among residents in aged care. Nutrients..

[52] Chen L-K, Liu L-K, Woo J, Assantachai P, Auyeung T-W, Bahyah KS (2014). Sarcopenia in Asia: consensus report of the Asian working Group for Sarcopenia. J Am Med Dir Assoc.

[53] Cruz-Jentoft AJ, Sayer AA, Schneider SM, Sieber CC, Topinkova E, Vandewoude M (2019). Sarcopenia: revised European consensus on definition and diagnosis. Age Ageing.

[54] Ge S, Du Q, Feng X, Liu Y, Wang H, Hai S (2022). Optimal cutoffs for the diagnosis of sarcopenia in older Chinese adults. Frontiers in Nutrition.

[55] Demling RH (2009). Nutrition, anabolism, and the wound healing process: an overview. Eplasty..

[56] Dzik KP, Kaczor JJ (2019). Mechanisms of vitamin D on skeletal muscle function: oxidative stress, energy metabolism and anabolic state. Eur J Appl Physiol.

[57] Chae SA, Kim H-S, Lee JH, Yun DH, Chon J, Yoo MC (2021). Impact of vitamin B12 insufficiency on sarcopenia in community-dwelling older Korean adults. Int J Environ Res Public Health.

[58] Fried LP, Tangen CM, Walston J, Newman AB, Hirsch C, Gottdiener J (2001). Frailty in older adults: evidence for a phenotype. J Gerontol A Biol Sci Med Sci.

[59] Landi F, Camprubi-Robles M, Bear DE, Cederholm T, Malafarina V, Welch AA (2019). Muscle loss: the new malnutrition challenge in clinical practice. Clin Nutr.

[60] Vandewoude MFJ, Alish CJ, Sauer AC, Hegazi RA (2012). Malnutrition-sarcopenia syndrome: is this the future of nutrition screening and assessment for older adults?. J Aging Res.

[61] Woo J (2018). Nutritional interventions in sarcopenia: where do we stand?. Curr Opin Clin Nutr Metab Care.

[62] Tan VMH, Pang BWJ, Lau LK, Jabbar KA, Seah WT, Chen KK (2021). Malnutrition and sarcopenia in community-dwelling adults in Singapore: Yishun health study. J Nutr Health Aging.

[63] Shaw SC, Dennison EM, Cooper C (2017). Epidemiology of sarcopenia: determinants throughout the lifecourse. Calcif Tissue Int.

[64] Du Y, Wang X, Xie H, Zheng S, Wu X, Zhu X (2019). Sex differences in the prevalence and adverse outcomes of sarcopenia and sarcopenic obesity in community dwelling elderly in East China using the AWGS criteria. BMC Endocr Disord.

[65] Lima RM, de Oliveira RJ, Raposo R, Neri SGR, Gadelha AB (2019). Stages of sarcopenia, bone mineral density, and the prevalence of osteoporosis in older women. Arch Osteoporos.

[66] Laskou F, Dennison E (2019). Interaction of nutrition and exercise on bone and muscle. Eur Endocrinol.

[67] Bauer J, Morley JE, Schols AMWJ, Ferrucci L, Cruz-Jentoft AJ, Dent E (2019). Sarcopenia: a time for action. An SCWD position paper J Cachexia Sarcopenia Muscle.

[68] Dent E, Morley JE, Cruz-Jentoft AJ, Arai H, Kritchevsky SB, Guralnik J (2018). International clinical practice guidelines for sarcopenia (ICFSR): screening, diagnosis and management. J Nutr Health Aging.

[69] Fagundes Belchior G, Kirk B, Pereira da Silva EA, Duque G (2020). Osteosarcopenia: beyond age-related muscle and bone loss. Eur Geriatr Med..

[70] Gielen E, Beckwee D, Delaere A, De Breucker S, Vandewoude M, Bautmans I (2021). Nutritional interventions to improve muscle mass, muscle strength, and physical performance in older people: an umbrella review of systematic reviews and meta-analyses. Nutr Rev.

[71] Izquierdo M, Merchant RA, Morley JE, Anker SD, Aprahamian I, Arai H (2021). International exercise recommendations in older adults (ICFSR): expert consensus guidelines. J Nutr Health Aging.

[72] Skipper A, Coltman A, Tomesko J, Charney P, Porcari J, Piemonte TA (2020). Adult malnutrition (undernutrition) screening: an evidence analysis center systematic review. J Acad Nutr Diet.

[73] Sergi G, De Rui M, Stubbs B, Veronese N, Manzato E (2017). Measurement of lean body mass using bioelectrical impedance analysis: a consideration of the pros and cons. Aging Clin Exp Res.

[74] Yamada Y, Nishizawa M, Uchiyama T, Kasahara Y, Shindo M, Miyachi M (2017). Developing and validating an age-independent equation using multi-frequency bioelectrical impedance analysis for estimation of appendicular skeletal muscle mass and establishing a cutoff for sarcopenia. Int J Environ Res Public Health.

[75] Buckinx F, Landi F, Cesari M, Fielding RA, Visser M, Engelke K (2018). Pitfalls in the measurement of muscle mass: a need for a reference standard. J Cachexia Sarcopenia Muscle.

[76] Fung FY, Koh YLE, Malhotra R, Ostbye T, Lee PY, Shariff Ghazali S (2019). Prevalence of and factors associated with sarcopenia among multi-ethnic ambulatory older Asians with type 2 diabetes mellitus in a primary care setting. BMC Geriatr.

[77] Lardiés-Sánchez B, Sanz-Paris A, Boj-Carceller D, Cruz-Jentoft AJ (2016). Systematic review: prevalence of sarcopenia in ageing people using bioelectrical impedance analysis to assess muscle mass. Eur Geriatr Med.

